# κ‑Ga_2_O_3_/(B)GaAs/GaAs Heterostructures: Study of Optically Active Defects, Design, and Modeling of Solar Cells Based on These Heterostructures

**DOI:** 10.1021/acsomega.5c09871

**Published:** 2025-12-12

**Authors:** Tarak Hidouri, Antonella Parisini, Babban Kumar Ravidas, Juan Jiménez, Dip Prakash Samajdar, Roberto Fornari

**Affiliations:** † Department of Mathematical Physical and Computer Sciences, 9370 University of Parma, Parma 43124, Italy; ‡ Department of Natural Sciences, 91563 PDPM IIITDM, Jabalpur, MP 482005, India; § GdS Optronlab, Department of Condensed Matter Physics, 16782 University of Valladolid, LUCIA Building, Paseo de Belen 19, Valladolid 47011, Spain; ∥ Department of Electronics and Communication Engineering, PDPM Indian Institute of Information Technology, Design and Manufacturing, Jabalpur 482005, India

## Abstract

This work focuses on the steady-state luminescence of pure-phase κ-Ga_2_O_3_/GaAs and κ-Ga_2_O_3_/BGaAs/GaAs heterostructures grown by metal–organic chemical vapor deposition. The films were characterized by electron beam (e-beam) energy-dependent cathodoluminescence (CL) and steady-state photoluminescence (PL), supported by one-dimensional Solar Cell Capacitance Simulator (SCAPS-1D) simulation, to assess their possible application as intermediate energy band layers in solar cells. The power- and temperature-dependent PL and CL show that the luminescence in κ-Ga_2_O_3_/GaAs and κ-Ga_2_O_3_/*a*-BGaO/BGaAs/GaAs heterostructures, where *a*-BGaO represents a thin amorphous BGaO interlayer between the κ-Ga_2_O_3_ film and BGaAs template, is dominated by donor–acceptor transitions. These transitions originated from point defect ensembles tuned by the disorder at the interface, which give rise to the formation of minibands. The boron-to-gallium substitution, boron segregation, gallium diffusion, and point defects clustering at the interface are supposed to play a crucial role in the luminescence mechanisms. The heterojunction features are promising in view of the development of a new class of active layers in photodetectors working in the visible and infrared regions. We propose a novel solar cell structure in which the insertion of a BGaAs/*a*-BGaO interlayer between GaAs and κ-Ga_2_O_3_ may substantially enhance the solar cell parameters. The SCAPS-1D simulations suggest that the κ-Ga_2_O_3_/*a*-BGaO/BGaAs/GaAs heterostructure may reach a power conversion efficiency (PCE) of 23.76% with an open-circuit voltage (*V*_oc_) of 0.92 V, a short-circuit current density (*J*_sc_) of 32.61 mA/cm^2^, and a fill factor (FF) of 78.66%.

## Graphical abstract

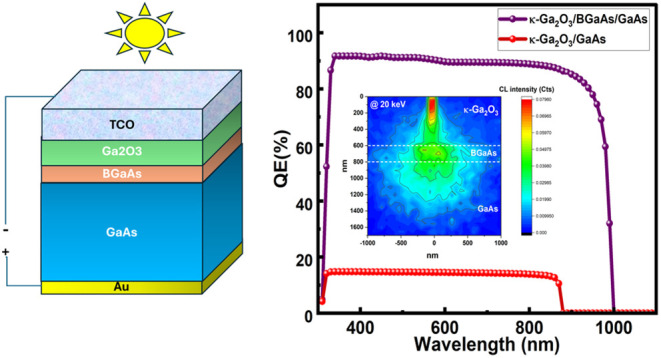

## 1 Introduction

The global shift toward renewable energy has driven intense research into advanced photovoltaic (PV) technologies capable of outperforming traditional single-junction solar cells (SCs). Among the most promising approaches, there is the intermediate band solar cell (IBSC), a concept proposed to overcome the Shockley–Queisser limit by enabling the absorption of low-energy (sub-bandgap) photons via an intermediate energy level forming an intermediate band (IB) positioned within the host semiconductor bandgap.^1,2^ This IB allows for two-step photon absorption: from the valence band (VB) to the IB, and from the IB to the conduction band (CB), thereby enhancing photocurrent generation while preserving high output voltage.^3^ Hence, the realization of efficient IBSCs requires advanced material systems with engineered band structures and tailored optical and electronic properties. A particularly attractive platform to achieve IBs involves wide/narrow bandgap heterostructures. The IB formation might be achieved at the interface via quantum confinement, defect states, or impurity-induced band anticrossing effects.^2^

In this context, gallium oxide (Ga_2_O_3_), a wide-bandgap oxide semiconductor with a bandgap in the range of 4.7–5.2 eV,^4,5^ depending on the considered polymorph, is gaining attention for its potential in both conventional and unconventional SC designs. Known for its high breakdown field (estimated to be ∼8 MV/cm), deep ultraviolet (UV) transparency up to the range UV–C, and excellent chemical stability, Ga_2_O_3_ has found applications in power electronics and solar-blind UV–C photodetectors.^4–6^ Recent studies also highlight its ability to form promising heterojunctions with semiconductors like GaAs, a well-established III–V material with a bandgap of 1.42 eV and high carrier mobility, suggesting the possibility of designing Ga_2_O_3_/GaAs heterostructures suitable for novel optoelectronic applications.^6,7^

Further tuning of the band structure and interfacial properties can be achieved by incorporating boron into GaAs, forming boron-doped and boron-alloyed GaAs (BGaAs).^8^ Boron atoms introduce localized discrete energy levels that interact with the CB of GaAs, effectively modifying the bandgap and the electron effective mass.^9^ Additionally, the addition of a BGaAs interlayer can reduce interface recombination and support sub-bandgap absorption pathways through localized states engineering critical for IBSC performance.

To evaluate the feasibility and performance of Ga_2_O_3_-on-III–V heterostructures, numerical simulations were carried out for predicting the device geometry prior to fabrication. The Solar Cell Capacitance Simulator, one-dimensional (SCAPS-1D), developed by the University of Ghent, is a widely used one-dimensional simulation program that solves Poisson’s and continuity equations under steady-state conditions. It has been extensively employed to simulate thin-film and multilayer SCs, including devices based on CdTe, CIGS, and perovskites.^10,11^ While SCAPS-1D was not originally designed for oxide/semiconductor or IB structures, recent works have demonstrated its suitability for modeling Ga_2_O_3_-based heterojunctions, oxide/III–V interfaces, and defect-assisted sub-bandgap transitions.^12–14^ To the best of our knowledge, there are no reports on SCAPS simulation or emission analysis of Ga_2_O_3_/GaAs or Ga_2_O_3_/BGaAs/GaAs heterostructures specifically designed for IBSCs. The existing studies primarily focus on Ga_2_O_3_/CdTe^14^ or Ga_2_O_3_/Cu_2_O interfaces,^12^ or experimental Ga_2_O_3_-based photodetectors,^15^ but none of them has addressed the optical emission behavior, possible integration in IB-based photovoltaic performances in κ-Ga_2_O_3_/p-GaAs or κ-Ga_2_O_3_/*a*-BGaO/BGaAs/p-GaAs architecture proposed in the present work. A recently published demonstration of epitaxial deposition of pure κ polymorph on p-GaAs wafers and on BGaAs/GaAs templates by Metal Organic Vapor Phase Epitaxy (MOVPE)^16^ provided an extensive overview of the structural, morphological, and interfacial properties.

In this study, we present a detailed investigation of the emission characteristics of such heterostructures through temperature-dependent PL and e-beam-energy-dependent CL to highlight the defect-assisted and radiative recombination mechanisms. Furthermore, the device performance of Ga_2_O_3_/GaAs and Ga_2_O_3_/BGaAs/GaAs heterostructures is predicted, specifically looking at their application in IBSCs. Band alignment and energy level formation at the interfaces, quantum efficiency (QE), and *J*–*V* characteristics under standard AM1.5 illumination are simulated using SCAPS-1D. This work aims at establishing the experimental and theoretical bases for the fabrication of Ga_2_O_3_-based IBSC architectures and offers novel insights into the role of oxide/III–V interfaces and boron incorporation in enabling high-performance SCs.

## 2 Experimental and Numerical Details

### 2.1 Growth of Heterostructures

Details about the growth, morphological, structural, and interfacial properties of κ-Ga_2_O_3_/GaAs and Ga_2_O_3_/BGaAs/GaAs were recently reported in ref 16. Briefly, Ga_2_O_3_ was deposited by metal–organic chemical vapor deposition (MOCVD) in a horizontal reactor at IMEM-CNR institute. Trimethylgallium (TMG) and pure water (H_2_O) were used as gallium and oxygen precursors, respectively, with dihydrogen (H_2_) used as a carrier gas. κ-Ga_2_O_3_ (600 nm) was deposited at 610 °C for 1 h on both p-type GaAs substrates and BGaAs/GaAs templates (thickness of BGaAs is ∼250 nm). BGaAs templates were deposited on (001) p-GaAs substrates by MOVPE at LMI laboratory, University Claude Bernard Lyon 1 (UCBL), using an atmospheric pressure T-shape horizontal reactor, at 500 °C, using diborane (B_2_H_6_), triethylgallium (TEG), and arsine (AsH_3_) as boron, gallium, and arsenic precursors, respectively, and H_2_ was used as a carrier gas. The atomic fraction of boron in the ternary alloy was in the range of 1.5–6%. Based on the comparative analysis presented in ref 16, the BGaAs template with a boron fraction of 2.2% was identified as the one offering the best combination of morphological, structural, and interfacial properties and was therefore selected for the present study.

### 2.2 Characterization and Simulation Details

The CL study was carried out at GdS Optronlab, Department of Condensed Matter Physics, University of Valladolid, with a MonoCL2 system from Gatan U.K., attached to a field emission scanning electron microscope (FESEM-LEO 1530). The detector for the spectral analysis was a Peltier-cooled CCD, while a photomultiplier was used for recording panchromatic CL images. CL spectra were acquired at room temperature. The e-beam energy varied between 5 and 30 keV, to change the probe depth. PL measurements were performed at UniPR using the green line of a continuous wave (CW) Ar^+^ laser (2.41 eV, 514.5 nm) with a maximum excitation power of 100 mW. PL measurements were carried out at low temperature (10 K), as a function of temperature (10–300 K), and by varying the excitation power, keeping samples in a closed-cycle helium cryostat. The emission was analyzed by a high-resolution spectrometer (Jobin-Yvon monochromator: focal length 0.6; resolution: 10 Å/mm; width of the input slit; two 600 lines/mm diffraction gratings). Detection was made by a phototube with a built-in amplifier (gain up to ∼10^4^).

SCAPS-1D software version 3.3.10, developed at the Department of Electronics and Information Systems, University of Gent, Belgium,^17^ was used for numerical simulations. It is based on the solution of the continuity equation and Poisson equation for electrons and holes. It enables an easy simulation of up to seven-layer SC under illumination and dark in the software ambient.^18^ The properties of the different layers were taken from previous literature and are listed in Table S1 (Supporting Information), while the interfacial parameters are listed in Table S6 (Supporting Information), both sets of parameters being necessary for simulation.

## 3 Results and Discussion

### 3.1 Emission Properties of κ-Ga_2_O_3_/p-GaAs

PL analysis was carried out to clarify the type and intensity of the optical transitions in GaAs and the way that they are influenced by the overlying Ga_2_O_3_. Figure 1a shows a broad emission that includes two contributions with a main emission centered at ∼1.475 eV (red-shifted of about 40 meV with respect to GaAs main peak) with a full width at half-maximum of 29 meV (typically ∼21 meV for GaAs). The asymmetric emission at the lower energy side (see the inset of Figure 1a) is reproduced, whatever the excitation power, which reflects its intrinsic origin. The GaAs substrate is p-type and contains a high density of shallow acceptor levels. Furthermore, it is conceivable that other acceptor levels form during the deposition of Ga_2_O_3_, either because of some Ga diffusion from the Ga_2_O_3_ film into the substrate (formation of Ga_As_ antisites) or due to the generation of new V_Ga_ in the substrate at the epitaxial growth temperature. Both types of point defects are recognized to behave as acceptors, but the literature is confused about their actual energy levels. At this stage, it is impossible to state whether the Ga_2_O_3_ deposition results in a Ga excess (Ga antisites) or deficiency (*V*_Ga_) in the GaAs substrate. The overall result is, however, that additional acceptors are created in the GaAs substrate, close to the heterojunction interface, upon epitaxy of Ga_2_O_3_, so that free electrons from the CB can recombine radiatively with holes trapped on these acceptors and produce the observed sub-bandgap PL transitions. Such acceptors can form a band-tail that is usually constituted by spatially close states in an ensemble, called point defect ensembles (PDEs), and their distribution leads to adjacent ensembles.^19,20^ This energy level configuration can facilitate the hopping and tunneling process between adjacent states in the same ensemble or between close PDEs.

**Figure 1:**
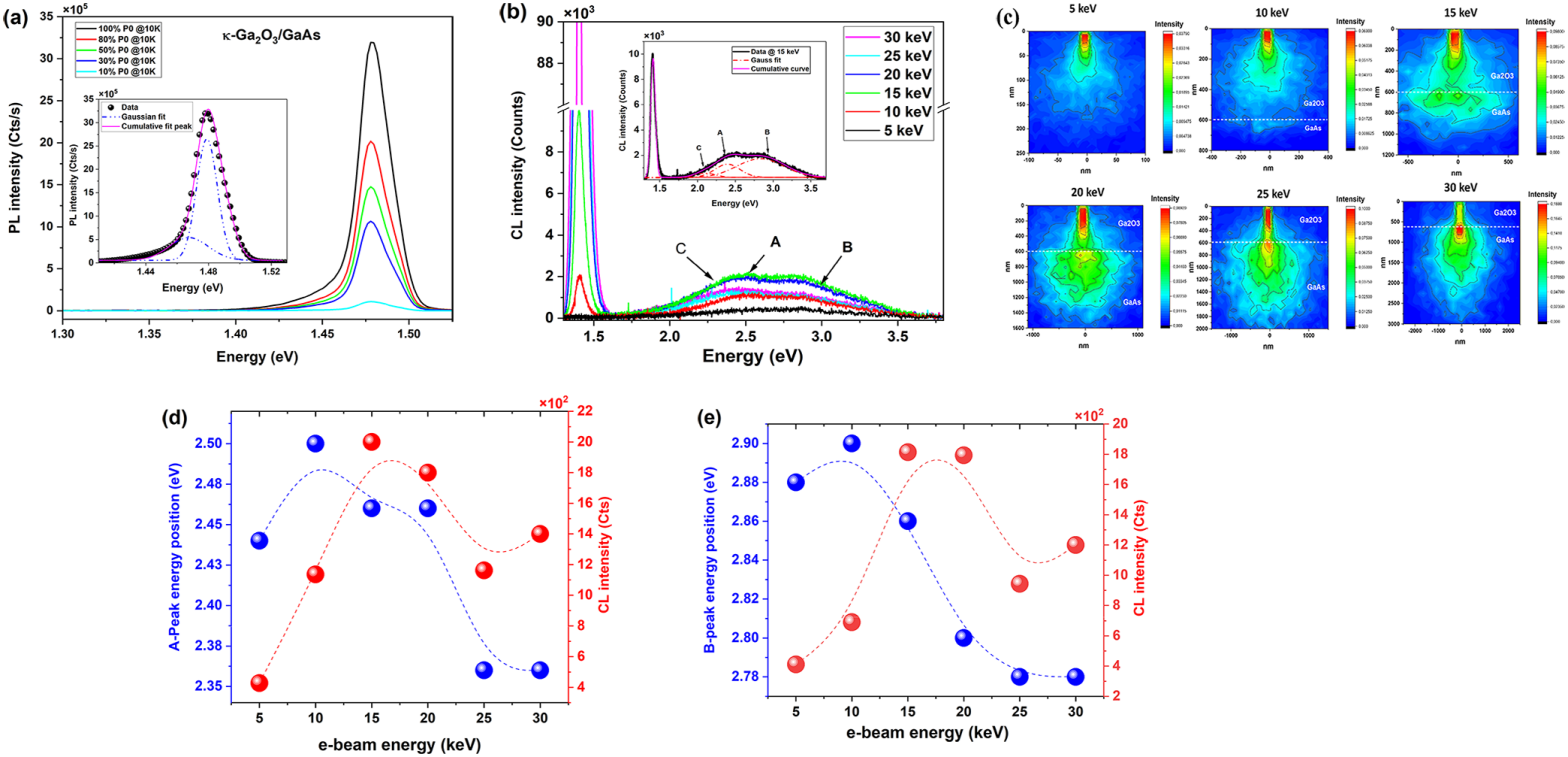
(a) Power-dependent PL spectra of κ-Ga_2_O_3_/GaAs at 10 K. The inset shows the 10 K PL spectrum recorded at a power P0 = 100 mW (black symbols) deconvoluted into two Gaussian peaks (dashed line); (b) CL spectra of κ-Ga_2_O_3_/GaAs vs e-beam energy at room temperature. The inset shows an example of a spectrum deconvolution (for 15 keV). (c) Results of the Casino simulation showing electron–hole (e–h) generation and diffusion volume at different e-beam energies. (d) Energy position of A peak and its corresponding CL intensity vs e-beam energy. (e) Energy position of B peak and its related CL intensity vs e-beam energy. Dashed lines in (d) and (e) are guides for the eyes.

To better understand the luminescence mechanisms, the power-dependent PL intensity of the main and low-energy peaks is reported in Figure S1 (Supporting Information). Both data can be described by a power law of the type *I*_PL_= α*P* ^*d*^, where *I*_PL_ is the PL intensity, *P* is the excitation power, α is a fitting parameter, and *d* is a factor that can take the following values 1 < *d* ≤ 2 for exciton-like transition while *d* ≤ 1 is attributed to free-to-bound transition or less probably to (*V*_As_–Ga_As_) donor–acceptor (D–A) pair transitions.^20–22^

The photogenerated electrons and holes in GaAs may diffuse in random directions, lose the correlation, and consequently recombine as free excitons (*d* = 2.05). On the other hand, the other slope (*d* = 0.63) indicates a recombination between the free-to-bound state and/or bound-to-bound state, precisely between free electrons to the mentioned energy levels, or donor-to-acceptor transitions, and then the luminescence is no more excitonic in nature. This phenomenon can be attributed to an enhancement of acceptor density in GaAs after depositing Ga_2_O_3_ as well as the interfacial oxygen that can be present at the GaAs/Ga_2_O_3_ interface and the related change in the density of states.^23,25^

CL versus e-beam energy (Figure 1b), supported by Casino simulations (Figure 1c), was performed at room temperature. At a low e-beam energy (5 keV), there is no emission from GaAs due to the insufficient e–h volume generation.^20^ However, for higher excitation energy, Figure 1b clearly shows an efficient emission from both GaAs and κ-Ga_2_O_3_ extended in the range of 1.3–3.54 eV (350–950 nm). The CL emission from Ga_2_O_3_ can be fitted with three Gaussian peaks labeled as A (2.43 eV), B (3.0 eV), and C (2.23 eV) (the inset of Figure 1b is taken as an example). These emissions fit well with those previously detected in κ-Ga_2_O_3_ films grown in H_2_ atmosphere^4,20^ that are extending over the photon energy range of 2–3.4 eV (360–620 nm) and composed of four bands centered at about 2.4, 2.75, 3.0, and 3.25 eV below CB.^4,20^ These peaks are always present, independently of the growth conditions; however, they exhibit significant differences in intensity and energy position probably due to defects and internal or external stress.^4,20^ The A and B peaks dominate the emission and are associated with *V*_Ga_-related defects, while the C-peak may be associated with oxygen vacancies (*V*_o_) and/or (*V*_o_–*V*_Ga_) complexes.^22,24^ The CL intensity increases, reaching its maximum at an e-beam energy of 15 keV, and then it starts dropping for higher e-beam energy. In fact, at this point, the e-beam can reach the GaAs layer (Figure 1c), achieving the largest excitation volume in Ga_2_O_3_, i.e., the highest density of photoexcited acceptors in this layer. After such e-beam energy, the e–h generation volume mainly extends into the GaAs substrate, and consequently, the emission from the Ga_2_O_3_ layer decreases, whereas the CL contribution from GaAs increases.

For both A and B peaks, the highlighted dependence of the CL intensity on the beam energy and the peak position is quantitatively reported in Figure 1d. A sudden blue shift of the energy position is followed by a red shift at higher excitation. Because A and B peaks are related to transitions involving *V*_Ga_ defects and their complexes, in particular hydrogenated *V*_Ga_,^18,21^ this result suggests that the beam energy increase favors the involvement of acceptors deeper into the bandgap. Considering the energy levels of hydrogenated *V*_Ga_ acceptors with a different number of incorporated H atoms (*n*H–*V*_Ga_, *n* = 1, 2, 3) reported in ref 21, one can think that the high-energy e-beam may break the complexes by removing one or more H atoms. The more H atoms are removed, the deeper the energy state. Also, the lattice distortion is expected to vary. As shown in ref 21, the energy level of pure *V*_Ga_ is about 2.5 eV, while complexes with 1, 2, 3 H atoms are located closer to VB, up to 1 eV, corresponding to full hydrogenation.

This phenomenology can produce a change in the aggregation of point defects and the depth of potential wells in which carriers are trapped. At low electron beam energy, we can refer to them as ensembles of shallower point defect complexes with weak carrier trapping (high *n*). For increasing electron beam energy, the complex defects will be progressively broken (progressive reduction of *n*), which results in deeper levels, with stronger carrier trapping, and finally to small point defect ensembles and red-shifted emission.^20^

On the other hand, the CL spectra show an enhanced contribution from GaAs, due to a higher e–h generation volume in this material. At the highest energy, GaAs emission is much higher than that of Ga_2_O_3_: *I*_*GaAs*_^30*keV*^/*I*_*Ga*2*O*3_^30*keV*^ = 75. In the CL process, the filling of the related acceptor states is allowed even at low e-beam excitation. Indeed, a monotonic increase of the CL emission of GaAs versus the beam energy can be seen in Figure 1b.

Figure 2 shows that the emission intensity of the low-energy PL peak energy associated with GaAs in κ-Ga_2_O_3_/GaAs heterojunction (red squares) decreases very rapidly versus the temperature up to 100 K. This can be attributed to the release of holes from acceptors located in the p-type substrate in the temperature range of 10–100 K. Compared with GaAs reference, the PL peak energy position shows a pronounced deviation from Varshni’s law when GaAs is overgrown by κ-Ga_2_O_3_, which supports the hypothesis that point defects and related acceptor levels are localized in the GaAs side of the heterostructure. With increasing thermal energy, carriers are more readily activated by a hopping process from families of vacancies to the VB, promoting a band-to-band transition. Such thermal quenching of the PL intensity starts at 90–100 K.

**Figure 2:**
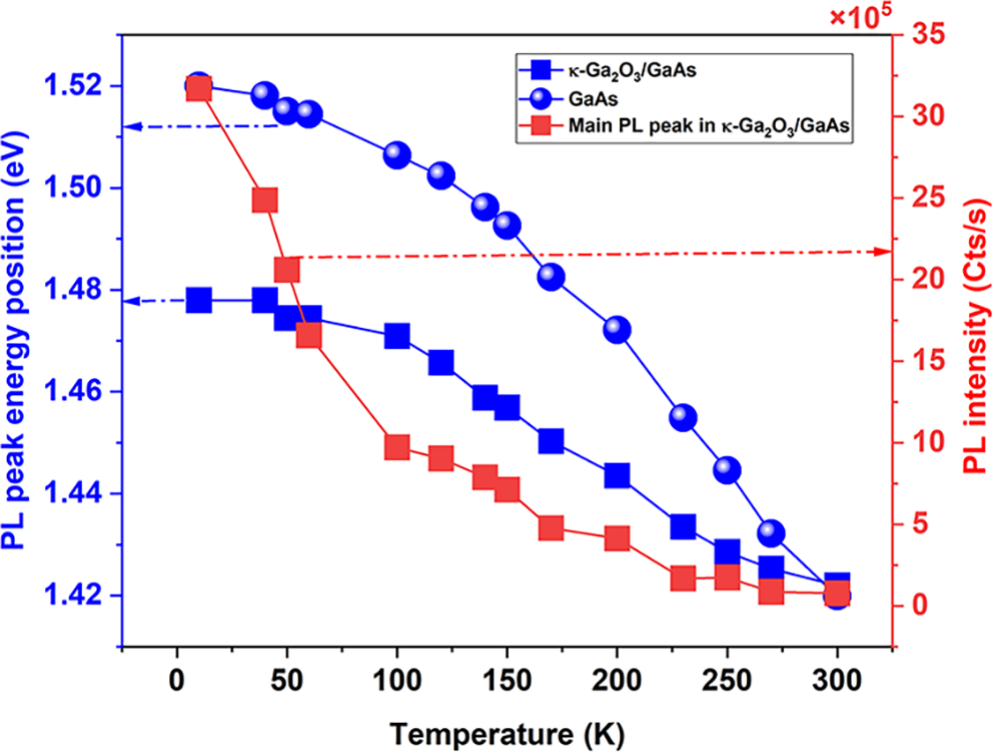
Temperature-dependent PL energy of the main emission peak of the undoped κ-Ga_2_O_3_/GaAs heterostructure (blue squares) and its corresponding intensity (red squares). The PL peak energy position of the GaAs reference substrate is also reported (blue balls). The excitation power is fixed at 100 mW for all temperatures.

### 3.2 Emission Properties of κ-Ga_2_O_3_/B_*x*_Ga_1–*x*_As/GaAs

A well-pronounced PL emission has been observed when a BGaAs interlayer between κ-Ga_2_O_3_ and GaAs epilayers is present. At low temperature, the PL spectrum (Figure 3a) can be fitted with three Gaussian peaks centered at 1.496 eV (∼829 nm), 1.470 eV (843 nm), and 1.458 eV (850.5 nm), denoted later as P1, P2, and P3, respectively.

**Figure 3:**
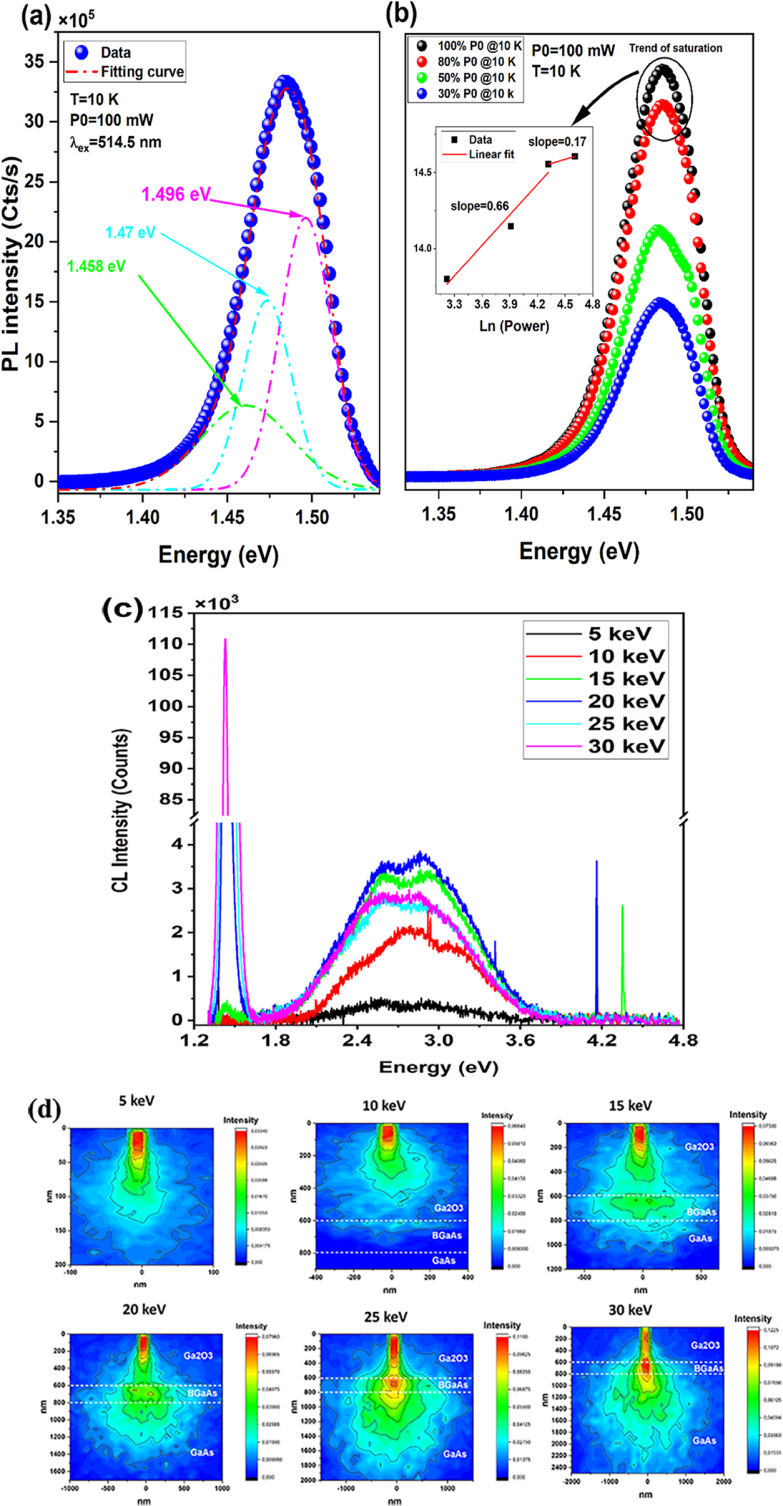
(a) 10 K PL spectrum of κ-Ga_2_O_3_/BGaAs/GaAs (black symbols) fitted with three Gaussian peaks (green solid line). (b) Power-dependent PL spectra at 10 K. Inset: The application of the power law for the PL intensity versus excitation power; the straight red lines express the best fits by the function *I*_PL_ = α*P* ^*d*^. (c) CL spectra versus e-beam energy. (d) The simulated e–h generation volume at different e-beam energies.

In BGaAs alloys, increasing the boron content to as much as 3% results in a blue shift of the PL emission compared with that of GaAs. However, in our structure, a pronounced boron segregation effect toward Ga_2_O_3_ has already been observed,^16^ leading to a gradient in the boron concentration within the BGaAs layer. Specifically, the boron content is minimal at the BGaAs/GaAs interface, which reduces the strain and increases toward the Ga_2_O_3_ layer. Based on this observation, the P2 peak can be attributed to emission from the BGaAs layer, exhibiting an asymmetric tail toward lower energies (P3). The latter, corresponding to the P3 peak, is associated with residual boron cluster states located near the CB of GaAs.^9,27^ In fact, despite boron segregation occurring at the surface of the BGaAs interlayer without boron evaporation,^27^ at the growth temperature of 610 °C, the residual boron in the ternary film forms clusters, thereby creating minibands near the CB minimum in the heterojunction and causing bandgap narrowing. The P1 peak, in turn, originates from the GaAs layer.

Photogenerated e–h carriers can be captured at localized minibands within the bandgap, which may be due to boron cluster states (near the CB minimum)^27^ and/or boron vacancies (*V*_B_) resulting from boron out-diffusion,^16^ or also *V*_Ga_ point defects (both near the VB maximum). The involvement in the PL process of similar defect states leads to emission saturation at high excitation power densities, exhibiting saturation-like behavior with a slope less than unity (the inset in Figure 3b). The power-law behavior shown in Figure 3b reveals two distinct regimes of sequential filling with increasing excitation power. This indicates that D–A transitions dominate the recombination mechanism at this temperature, involving at least two D–A families with different densities. Such inhomogeneity can be attributed to the presence of multiple *V*_Ga_ families randomly distributed throughout the material. The substitution of boron by gallium and the filling of *V*_Ga_ sites by boron atoms are highly probable. Consequently, the spatially inhomogeneous distribution of *V*_Ga_ may be easily coupled to boron segregation and the resulting boron clustering. The observed saturation at high excitation powers (the inset of Figure 3b) reflects carrier localization induced by disordered boron clusters, *V*_B_, and PDEs, which form band-tail states capable of trapping excitons.

CL measurements and the Casino simulation reported in Figure 3c,d confirm the previous findings. Indeed, the heterostructure exhibits a continuous increase in CL intensity with increasing electron beam energy (from 5 to 20 keV), followed by saturation at high excitation levels and a subsequent decrease in intensity at 25 and 30 keV. The overall e–h generation volume is larger in the κ-Ga_2_O_3_/BGaAs/GaAs heterostructure than in the Ga_2_O_3_/GaAs reference, resulting in enhanced emission.

By introducing the BGaAs interlayer, a clear enhancement of the CL emission in the visible range is observed compared with the reference κ-Ga_2_O_3_/GaAs heterostructure (Figure S2, Supporting Information) even at low beam energies. It is worth recalling that the excess segregated boron on the top surface of the BGaAs interlayer reacts with gallium and oxygen during the Ga_2_O_3_ growth process, forming a thin amorphous layer, likely gallium borate (BGaO_3_), at the BGaAs/Ga_2_O_3_ interface, as confirmed by TEM analysis in ref 16. The formation of this thin amorphous BGaO interlayer on top of the BGaAs template is believed to contribute to the observed emission enhancement. In particular, the boron excess may react with gallium and oxygen at the very initial stage of Ga_2_O_3_ deposition, thereby preventing arsenic diffusion, as demonstrated in ref 16. In addition, the lattice mismatch is reduced,^16^ which decreases the probability of nonradiative recombination at the Ga_2_O_3_–BGaO interface, thereby enhancing the CL emission from Ga_2_O_3_. A comparison of the CL spectra from both structures reveals a slight blue shift in the infrared region, which can be attributed to the strain relaxation and the reduced concentration of *V*_Ga_ resulting from boron atoms occupying gallium sites.

The temperature-dependent PL spectrum is characterized by two primary emissions, P1 and P2, whose positions and intensities vary with the temperature. At cryogenic temperatures, both peaks exhibit high intensities due to the increased localization of carriers, which leads to stronger radiative recombination (see the inset in Figure 4a). As the temperature rises, P2 decreases monotonically up to 120 K, and then it undergoes complete quenching. In contrast, P1 remains across the entire temperature range. Between 10 and 120 K, carriers are thermally excited out of localized states and can transition to higher energy states, resulting in a blue shift in the emission. This is accompanied by a decrease in emission intensity due to enhanced nonradiative recombination.^19^ As the temperature continues to increase, carriers gain sufficient thermal energy to overcome small potential barriers, moving to higher energy states. Above 120 K, P2 disappears as carriers become free to recombine after moving from the CB minimum and/or shallow donors to shallow acceptors near VB. Meanwhile, P1 persists, and its temperature-dependent behavior follows the classical Varshni’s model of bandgap shrinkage.

**Figure 4:**
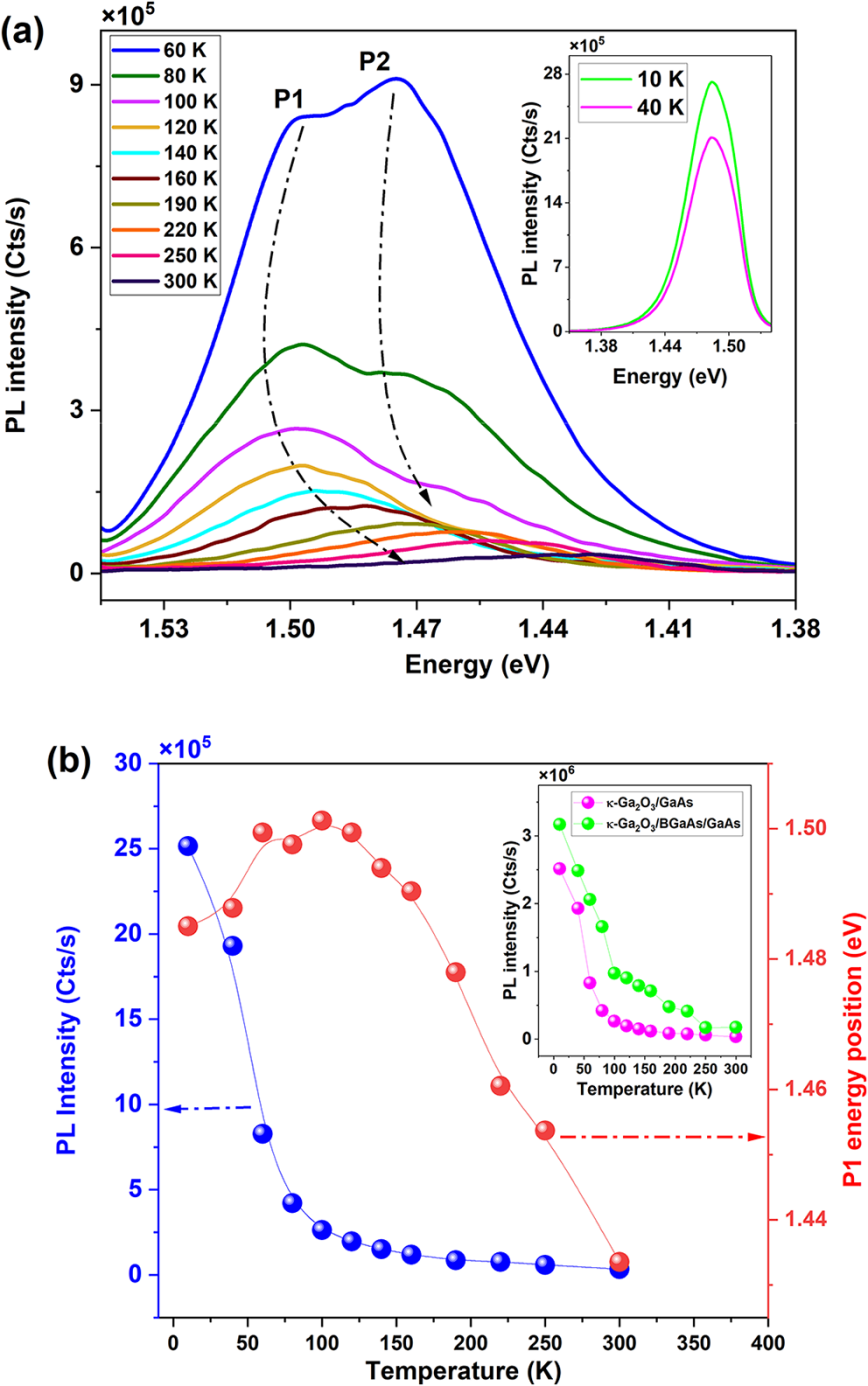
(a) Temperature-dependent PL of κ-Ga_2_O_3_/BGaAs/GaAs. Inset: The selected low-temperature PL spectra were recorded at 10 and 40 K. (b) P1 energy position (red symbols) and the corresponding PL intensity (blue symbols) as a function of temperature. Inset: Comparison of PL intensity quenching between κ-Ga_2_O_3_/BGaAs/GaAs and κ-Ga_2_O_3_/GaAs.

When compared with the κ-Ga_2_O_3_/GaAs structure, the PL intensity of the κ-Ga_2_O_3_/BGaAs structure decreases more rapidly with increasing temperature (the inset in Figure 4), primarily due to charge transfer. This process makes nonradiative recombination easier, but it facilitates more efficient charge transfer. Specifically, the defective κ-Ga_2_O_3_/BGaAs interface reduces PL emission, likely due to strong electron–phonon coupling with defects. Nonetheless, despite these effects, the κ-Ga_2_O_3_/BGaAs/a-BGaO/GaAs structure still exhibits appreciable emission at room temperature, in contrast to the κ-Ga_2_O_3_/GaAs structure.

## 4 Design and Simulation of a Solar Cell Based on κ-Ga_2_O_3_/(B)GaAs/GaAs

Based on the properties of κ-Ga_2_O_3_/BGaAs/GaAs, we designed an SC prototype (Figure 5a). Numerical details as well as the effect of varying the different parameters that affect the SC performance are provided in the Supporting Information. Figure 5a shows the schematic of the proposed SC. The device features a transparent conducting oxide (TCO) anode (work function: 3.8 eV) and a gold (Au) back contact cathode (work function: 5.1 eV). Ga_2_O_3_ serves as the n-type layer, the BGaAs interlayer serves as the absorber, and the p-type GaAs substrate acts as the hole transport layer. The results are obtained for one sun illumination conditions. Other working conditions considered for device simulation include a temperature of 300 K, series resistance of 2 Ω•cm^2^, shunt resistance of 10^4^ Ω·cm^2^, and TCO transmission spectrum equal to solar glass. The band-to-band radiative and Auger recombination are also considered to make the SC simulation replicate the practical results. Owing to the amorphous nature of the BGaO interlayer and the limited thickness, direct optical characterization could not be performed. In the SCAPS-1D simulations, this transition region was represented implicitly through consideration of a moderate conduction band offset (CBO) between κ-Ga_2_O_3_ and BGaAs. The defect density near the interface was set at 1 × 10^14^ cm^–3^. This modeling approach captures the electronic influence of the amorphous BGaO layer on charge transport and junction recombination without requiring explicit representation of an additional amorphous sublayer.

**Figure 5:**
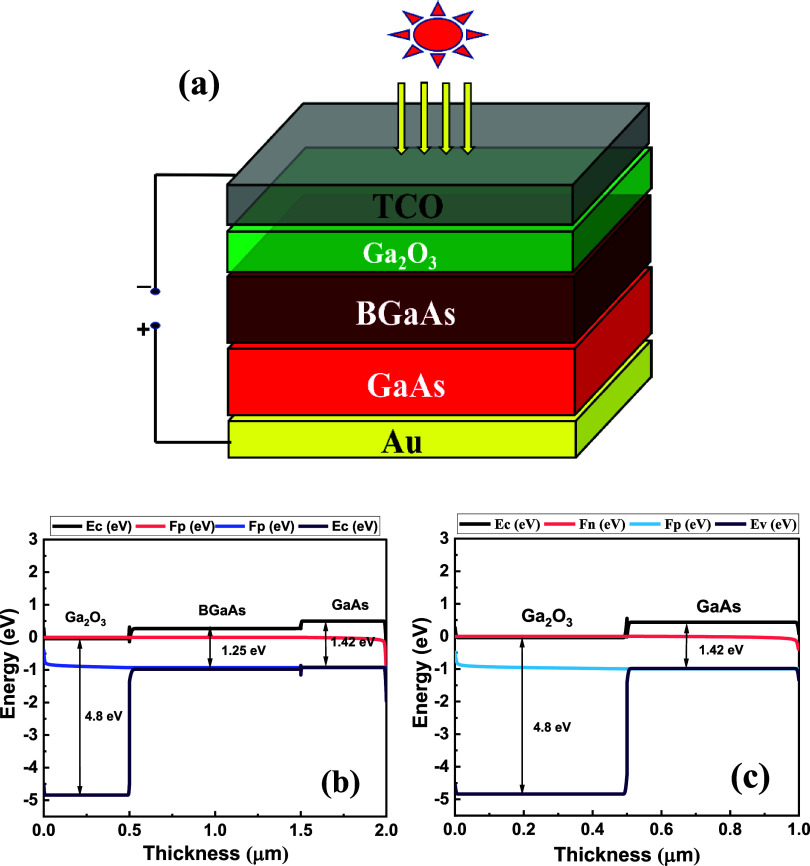
(a) Schematic of the proposed SC structure. (b) Simulated band offset diagram of TCO/Ga_2_O_3_/BGaAs/GaAs/Au. (c) Simulated band offset diagram of TCO/Ga_2_O_3_/GaAs/Au.

Considering the band offset of the three active materials in Figure 5a, the energy band diagrams of TCO/Ga_2_O_3_/BGaAs/GaAs/Au and TCO/Ga_2_O_3_/GaAs/Au devices are illustrated in Figure 5b,c. The band alignment between different layers is crucial for efficient transportation of photogenerated charge carriers from the absorber to respective electrodes. The addition of a BGaAs layer between p-GaAs and κ-Ga_2_O_3_ layers results in a decrease in the conduction band offset (CBO) and an increase in the valence band offset (VBO), which is favorable for charge transport inside the cell.

CBO/VBO at the interface of Ga_2_O_3_/BGaAs and BGaAs/GaAs are 0.45/3.1 eV and 0.12/0.29 eV, respectively. While in the BGaAs-free device, the CBO/VBO at the interface of Ga_2_O_3_/GaAs is 0.57/2.81 eV. *J*–*V* and QE of TCO/Ga_2_O_3_/BGaAs/GaAs/Au and TCO/Ga_2_O_3_/GaAs/Au configurations demonstrate the pronounced effect of inserting the BGaAs interlayer, as shown in Figure 6a,b.

**Figure 6:**
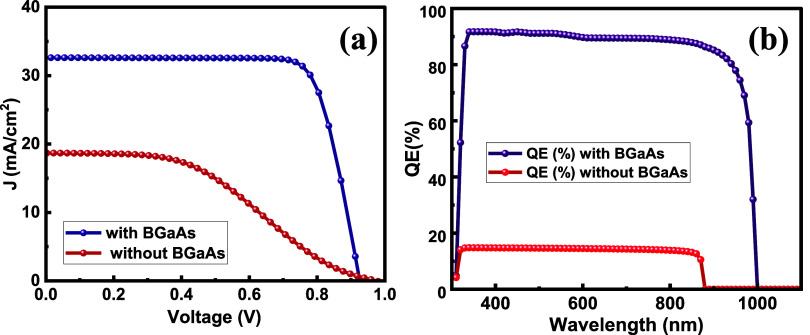
(a) Simulated *J*–*V* curves and (b) the simulated QE of the proposed prototypes based on TCO/κ-Ga_2_O_3_/BGaAs/GaAs/Au and TCO/κ-Ga_2_O_3_/GaAs/Au.

Under forward bias, the natural built-in electric field (from p-GaAs to n-Ga_2_O_3_) opposes carrier flow. Applying a forward bias reduces this potential, allowing carriers to move more freely. As the CB edge increases from Ga_2_O_3_ to BGaAs to GaAs, electrons from Ga_2_O_3_ are blocked by the higher CB of BGaAs and allow smooth electron flow from BGaAs to Ga_2_O_3_. Such band engineering results in the suppression of electron injection. This is like the “blocking” function of the n–i–p structure, and one can assign the BGaAs layer as a CB barrier. However, the VB barrier between p-GaAs and BGaAs is much smaller, selectively allowing holes (majority carriers in p-GaAs) to overcome this small barrier and reach Ga_2_O_3_ under forward bias. This will result in a more efficient collection of photogenerated holes and suppress electron leakage, especially thermionic or tunneling currents. Moreover, BGaAs provides an ideal bandgap for the SC as an interlayer absorber in TCO/Ga_2_O_3_/BGaAs/GaAs/Au-based SC due to its superior photon absorption capability for photovoltage generation. According to the Shockley–Queisser limit of the single-junction SC, materials with bandgaps ranging from 1.1 to 1.5 eV provide maximum photocurrent and photovoltage, resulting in higher theoretical PCE. For this range of bandgaps, the material layer absorbs a substantial portion of the visible and near-infrared solar spectrum.

The PCE, *J*_sc_, *V*_oc_, and FF of the proposed prototype have been optimized by varying the bandgap, electron affinity, layer thickness, bulk defect density, and the acceptor doping density in the BGaAs layer (Figures S3–S7, Supporting Information). However, when using the input parameters of Tables S1 and S6, except for the donor density and electron mobilities in Ga_2_O_3_ (Tables S2 and S5, Supporting Information), it seems that the acceptor density and deep defect density do not change the SC performance (Tables S3 and S4, Supporting Information). In the proposed cell structure, comparing the output parameters in Table 1, the insertion of the BGaAs template between p-GaAs and κ-Ga_2_O_3_ enhances the SC properties, giving a PCE = 23.76%, *V*_oc_ = 0.92 V, *J*_sc_= 32.61 mA/cm^2^, and an FF = 78.66%. The cell without BGaAs exhibits inferior performance, with PCE = 7.49%, *V*_oc_ = 0.97 V, *J*_sc_= 18.67 mA/cm^2^, and FF = 41.15%.

**Table 1:** Parameters Deduced from Calculated *J*–*V* Curves for Both Device Structures

Device structure	*J*_sc_ (mA/cm^2^)	V _oc_ (V)	FF (%)	PCE (%)
TCO/Ga_2_O_3_/GaAs/Au	18.67	0.97	41.15	7.49
TCO/Ga_2_O_3_/BGaAs/GaAs/Au	32.61	0.92	78.66	23.76

The prototype proposed in this work shows a higher PCE than other previous structures, such as the graphene/GaAs SC (8.63%), graphene/h-BN/GaAs (10.18%),^28,29^ and MoS_2_/h-BN/GaAs SCs (9.03%),^30^ and also higher than those lithographically fabricated, such as Pt/BaTiO_3_/β-Ga_2_O_3_ (14.5%),^31^ planar NiO/*k*-Ga_2_O_3_/Al_2_O_3_, and vertical NiO/Ga_2_O_2_/β-Ga_2_O_3_/Si.^32,33^ Interestingly, the BGaAs/κ-Ga_2_O_3_-based device achieved a QE of around 90%, compared with 15% with the device without BGaAs. This result shows that the generation and collection of charge carriers are improved after the inclusion of BGaAs in the SC. Such an enhancement is because of the role of the BGaAs interlayer in manipulating carrier transport across the junction. Hence, in the conventional GaAs/κ-Ga_2_O_3_ p–n heterojunction, thermionic emission of electrons from the n-side to the p-side can lead to a high dark current, which degrades the photovoltaic performance. However, the BGaAs barrier layer introduces a CBO, which blocks electron injections from n-Ga_2_O_3_ into p-GaAs (this can happen even under reverse bias or at zero bias and dark conditions for photodetectors) but allows hole transport from p-GaAs to n-Ga_2_O_3_. Photogenerated holes in the p-GaAs layer can still tunnel or thermally hop through the barrier and be collected on the n-side. This leads to a lower dark current, improving the *V*_oc_ and overall efficiency. The BGaAs can constitute a thin barrier layer that helps in reducing interface trap states and recombination losses that improve FF, which, in turn, improves the carrier lifetime and the collection efficiency. One must consider that the thermal stability and long-term performance of the Ga_2_O_3_/BGaAs/GaAs heterostructures are mainly influenced by the amorphous BGaO interlayer and the interfaces. Amorphous oxides can partially crystallize or undergo phase separation at elevated temperatures (>400 °C), introducing trap states and affecting carrier transport. Diffusion of B, Ga, or O across interfaces may modify band alignment, increasing recombination and reducing *V*_oc_. Photoinduced defect generation, hydrogen migration affecting *n*H–V_Ga_ complexes, and mechanical stress from thermal cycling may further contribute to degradation. SCAPS simulations do not capture these effects, but extrapolation from similar structures suggests good stability under typical operating conditions (<100 °C).

## 5 Conclusion

κ-Ga_2_O_3_/GaAs and κ-Ga_2_O_3_/BGaAs/GaAs heterostructures, prepared by MOCVD, were characterized by PL and CL. Low-temperature PL measurements of κ-Ga_2_O_3_/GaAs structure evidenced a point defect-related luminescence, a broadening, and a red shift of the buried GaAs layer with respect to pristine GaAs. This is believed to be related to the diffusion of Ga atoms from the top κ-Ga_2_O_3_ layer into the GaAs substrate, which reduces the vacancy density and generates Ga_As_ acceptors.

PL and CL measurements show that luminescence in buried GaAs is not of an excitonic nature and more likely dominated by donor-to-acceptor transitions related to *V*_Ga_ that form PDEs distributed in the bandgap. The observations made on κ-Ga_2_O_3_/GaAs are useful to understand the luminescence mechanisms in the κ-Ga_2_O_3_/BGaAs/GaAs heterostructure. In the latter case, the CL intensity is double in comparison to that of κ-Ga_2_O_3_/GaAs, with the emission tuned by PDEs forming band-tail states.

Based on the properties of the *κ-*Ga_2_O_3_/BGaAs/GaAs heterostructure, we proposed a SC prototype that includes this material stack. The simulation of the TCO/Ga_2_O_3_/BGaAs/GaAs/Au prototype suggests a total QE of ∼90%, six times higher than TCO/Ga_2_O_3_/GaAs/Au and a broadened response ranging from the deep UV to the infrared spectrum (200–1000 nm). The insertion of a BGaAs interlayer in the SC suggests a PCE of 23.76%, *V*_oc_ of 0.92 V, *J*_sc_ of 32.61 mA/cm^2^, and an FF of 78.66%. This preliminary demonstration of the κ-Ga_2_O_3_/(B)­GaAs/GaAs heterostructure is expected to pave the way to functional and physical integration of Ga_2_O_3_ with other materials. The fabrication of fully functional devices and their testing, including accelerated aging, thermal cycling, electrical characterization after prolonged illumination, and bias stress tests, combined with structural and compositional analyses, are necessary in order to quantify degradation mechanisms and confirm the long-term stability of the heterostructures.

## Supplementary Material



## Notes

Power law of GaAs and κ-Ga_2_O_3_/GaAs (Figure S1); Comparison of the CL emission from κ-Ga_2_O_3_/GaAs and κ-Ga_2_O_3_/BGaAs/GaAs heterostructures at different e-beam energies (Figure S2); Effect of the BGaAs bandgap variation on the SC performance parameters (Figure S3); Analysis of the electron affinity effect in the BGaAs layer on the PCE, FF, *J*_sc_, and *V*_oc_ (Figure S4); Effect of the BGaAs thickness on the PCE, FF, *J*_sc_, and *V*_oc_ (Figure S5); Effect of the bulk defect density in the BGaAs layer on the PCE, FF, *J*_sc_, and *V*_oc_ (Figure S6); Effect of the acceptor defect density in the BGaAs layer on the PCE, FF, *J*_sc_, and *V*_oc_ (Figure S7); Input parameters of the different PSC layers used in SCAPS-1D simulation (Table S1); Variation of donor and acceptor density in Ga_2_O_3_ and its effect on the cell performance parameters (Tables S2 and S3); Deep trap center density variation of Ga_2_O_3_ layer and its effect on the SC performances (Table S4); Effect of varying the electron mobility of Ga_2_O_3_ on the SC performances (Table S5); Input parameters of the interface layer for SCAPS-1D simulation (Table S6) (PDF)

## References

[1] Luque A., Antonio M., Colin S. (2012). Understanding intermediate-band solar
cells. J. Nat. Photonics.

[2] Walukiewicz W., Shan W., Yu K. M., Ager J. W., Haller E. E., Miotkowski I., Seong M. J., Alawadhi H., Ramdas A. K. (2000). Interaction of localized electronic states with the
conduction band: Band anticrossing in II–VI semiconductor ternaries. Phys. Rev. Lett..

[3] Okada Y., Ekins-Daukes N. J., Kita T., Tamaki R., Yoshida M., Pusch A., Hess O., Phillips C. C., Farrell D. J., Yoshida K., Ahsan N., Shoji Y., Sogabe T., Guillemoles J. F. (2015). Intermediate band solar cells: Recent
progress and
future directions. Appl. Phys. Rev..

[4] Montedoro V., Torres A., Dadgostar S., Jimenez J., Bosi M., Parisini A., Fornari R. (2021). Cathodoluminescence
of undoped and
Si-doped ε-Ga_2_O_3_ films. Mater. Sci. Eng., B.

[5] Yuehui W., Yuqing T., Haoran L., Zhibin Y., Qingyi Z., Zhenbei H., Xu H., Xianhua W., Weihua T., Wen H., Zhenping W. (2021). p-GaSe/n-Ga_2_O_3_ van der Waals
heterojunction photodetectors. ACS Photonics.

[6] ana, S. ; Sergii, K. ; Ihor, B. ; Ivan, K. ; Dariya, D. ; Anatoli, I. P. In Ga2O3/por-GaAs/mono-GaAs heterostructures for advanced electronic applications, Proc. IEEE 7th Int. Conf. Smart Technol. Power Eng. Electron. (STEE); IEEE, 2024; pp TT3.25.1–TT3.25.4 10.1109/STEE63556.2024.10747888.

[7] Alfred M., Saqib R., Ciaran L., Dan L., Lijie L. (2025). A review of
Ga_2_O_3_ heterojunctions for deep-UV photodetection:
Current progress, methodologies, and challenges. Adv. Electron. Mater..

[8] Feodosyev S. B., Sirenko V. A., Syrkin E. S., Manzhelii E. V., Bondar I. S., Minakova K. A. (2023). Localized and quasi-localized
energy
levels in the electron spectrum of graphene with isolated boron and
nitrogen substitutions. Low Temp. Phys..

[9] Qian M., Rasha H. E., Corey W. R., Tuhin D., Shamim R. M., Seth R. B., Mark A. W. (2022). Effects
of B and In on the band structure
of BGa­(In)As alloys. J. Appl. Phys..

[10] Sharma A. (2020). Theoretical
investigation of BGaAs/GaAs for optoelectronic device applications. J. Electron. Mater..

[11] Burgelman M., Nollet P., Degrave S. (2000). Modeling polycrystalline semiconductor
solar cells. Thin Solid Films.

[12] Chua D., Kim S. B., Gordon R. (2019). Enhancement
of the open circuit voltage
of Cu_2_O/Ga_2_O_3_ heterojunction solar
cells through the mitigation of interfacial recombination. AIP Adv..

[13] Yakimov E. B., Yakimov E. E., Polyakov A. Y., Vasilev A. A., Schemerov I. V., Kuznetsov A., Pearton S. J. (2025). Depth-resolved cathodoluminescence
in γ/β-Ga_2_O_3_ polymorph junctions. APL Mater..

[14] Mouhammed A. A., Saleh A. N. (2019). SCAPS modeling of
Ga_2_O_3_/CdTe
heterojunction. Tikrit J. Pure Sci..

[15] Jiang M., Golovynskyi S., Chen J., Yang Z., Lv T., Huang G., Sun Z., Li L., Wu H., Li B. (2025). High-sensitive solar-blind
β-Ga_2_O_3_ thin-film
photodetector deposited by PLD optimizing growth temperature. Vacuum.

[16] Hidouri T., Nasi L., Ferrari C., Ferrari E., Bosi M., Rodriguez P., Seravalli L., Pedrielli A., Fornari R. (2025). Single-phase κ-Ga_2_O_3_ films
deposited by metal–organic chemical vapor deposition on GaAs
and ternary B_x_Ga_1–x_As templates. Appl. Surf. Sci..

[17] El-Naggar A. A., Eid A. M., Rafat Y., Khamis M. A., Bakry M., Elkun S., Ismail W., Sharshir S. W., El-Shaer A., Abdelfatah M. (2025). SCAPS simulation and design of highly
efficient CuBi_2_O_4_-based thin-film solar cells
(TFSCs) with hole
and electron transport layers. Sci. Rep..

[18] Hidouri T., Rabhi S., Bencherif H., Fornari R. (2025). Design of CsSnBr_3_/Ga_2_O_3_ hybrid photodetectors for high
UV selectivity and bifacial usage. Adv. Theory
Simul..

[19] Bhuyan S., Das S. K., Dhar S., Pal B., Bansal B. (2014). Optical density
of states in ultradilute GaAsN alloy: Coexistence of free excitons
and impurity band of localized and delocalized states. J. Appl. Phys..

[20] Hidouri T., Parisini A., Dadgostar S., Jimenez J., Fornari R. (2022). Point defect
localization and cathodoluminescence emission in undoped κ-Ga_2_O_3_. J. Phys. D: Appl. Phys..

[21] Mazzolini P., Varley J. B., Parisini A., Sacchi A., Pavesi M., Bosio A., Bosi M., Seravalli L., Janzen B. M., Marggraf M. N., Bernhardt N., Wagner M. R., Ardenghi A., Bierwagen O., Falkenstein A., Kler J., De Souza R. A., Martin M., Mezzadri F., Borelli C., Fornari R. (2024). Engineering shallow
and deep level defects in κ-Ga_2_O_3_ thin
films: Comparing metal-organic vapour phase epitaxy to molecular beam
epitaxy and the effect of annealing treatments. Mater. Today Phys..

[22] Dittrich Th., Parisini A., Pavesi M., Baraldi A., Sacchi A., Mezzadri F., Mazzolini P., Bosi M., Seravalli L., Bosio A., Fornari R. (2024). Electronic
states near surfaces and
interfaces of β-Ga_2_O_3_ and κ-Ga_2_O_3_ epilayers investigated by surface photovoltage
spectroscopy, photoconductivity and optical absorption. Surf. Interfaces.

[23] Holland M., Stanley C. R., Reid W., Hill R. J. W., Moran D. A. J., Thayne I., Paterson G. W., Long A. R. (2007). Ga_2_O_3_ grown on GaAs by molecular beam epitaxy for
metal oxide semiconductor
field-effect transistors. J. Vac. Sci. Technol.
B.

[24] Robertson J. (2009). Model of interface
states at III–V oxide interfaces. Appl.
Phys. Lett..

[25] Penman L. T., Johnston Z. M., Edwards P. R., Oshima Y., McAleese C., Mazzolini P., Bosi M., Seravalli L., Fornari R., Martin R. W., Massabuau F. C.-P. (2025). Comparative
study of the optical properties of α-, β-, and κ-Ga_2_O_3_. Phys. Status Solidi B.

[27] Rodriguez P., Auvray L., Favier A., Dazord J., Monteil Y. (2008). Influence
of boron surface enrichment on the growth mode of BGaAs epilayers
grown on GaAs by metalorganic vapour phase epitaxy. Thin Solid Films.

[28] Li X., Chen W., Zhang S., Wu Z., Wang P., Xu Z., Chen H., Yin W., Zhong H., Lin S. (2015). 18.5% efficient
graphene/GaAs van der Waals heterostructure solar cell. Nano Energy.

[29] Li X., Lin S., Lin X., Xu Z., Wang P., Zhang S., Zhong H., Xu W., Wu Z., Fang W. (2016). Graphene/h-BN/GaAs
sandwich diode as solar cell and photodetector. Opt. Express.

[30] Lin S., Li X., Wang P., Xu Z., Zhang S., Zhong H., Wu Z., Xu W., Chen H. (2015). Interface-designed MoS_2_/GaAs heterostructure solar cell with sandwich-stacked hexagonal
boron nitride. Sci. Rep..

[31] Wriedt N., Meng L., Yu D. S., Chae C., Liddy K., Dheenan A., Dhara S., Myers R. C., Maksimov O., Blakeley R., Krishna S., Hwang J., Zhao H., McGlone J., Rajan S. (2025). Enhanced UV–Vis rejection
ratio in metal/BaTiO_3_/β-Ga_2_O_3_ solar-blind photodetectors. Adv. Electron.
Mater..

[32] Moumen A., Rajabi Kalvani P., Matteia F., Foti G., Parisini A., Mosca R., Pavesi M., Bosi M., Seravalli L., Mezzadri F., Baraldi A., Mazzolini P., Vantaggio S., Bosio A., Fornari R. (2025). Self-powered NiO/κ-Ga_2_O_3_ heterojunction photodiode for fast broadband
ultraviolet (UV) radiation detection. Opt. Mater..

[33] Manuel J., Vasquez T., Ashai A., Lu Y., Khandelwal V., Rajbhar M., Kumar M., Li X., Sarkar B. (2023). A self-powered
and broadband UV PIN photodiode employing a NiO_x_ layer
and a β-Ga_2_O_3_ heterojunction. J. Phys. D: Appl. Phys..

