# Supplementary material for: κ‑Ga2O3/(B)GaAs/GaAs Heterostructures: Study of Optically Active Defects, Design, and Modeling of Solar Cells Based on These Heterostructures
Source: ACS Omega. 2025 Dec 12;10(51):63349–58. doi: 10.1021/acsomega.5c09871 (PMC12756735; doi:10.1021/acsomega.5c09871)
Supplement: Supplementary file 1 [file ao5c09871_si_001.pdf]

Supporting Information

κ-Ga_2O_3/(B)GaAs/GaAs heterostructures: study of optically active defects, design and modelling of solar cells based on these heterostructures

Tarak Hidouri[1], Antonella Parisini[1], Babban Kumar Ravidas[2], Juan Jiménez[3], Dip Prakash Samajdar[4], Roberto Fornari[1]

[1]Department of Mathematical Physical and Computer Sciences University of Parma, Parma 43124, Italy

[2]Department of Natural Sciences, PDPM IIITDM Jabalpur, MP-482005, India

[3]GdS Optronlab, Department of Condensed Matter Physics, University of Valladolid, LUCIA Building, Paseo de Belen 19, Valladolid, 47011, Spain

[4]Department of Electronics and Communication Engineering, PDPM Indian Institute of Information Technology, Design and Manufacturing, Jabalpur, 482005, India

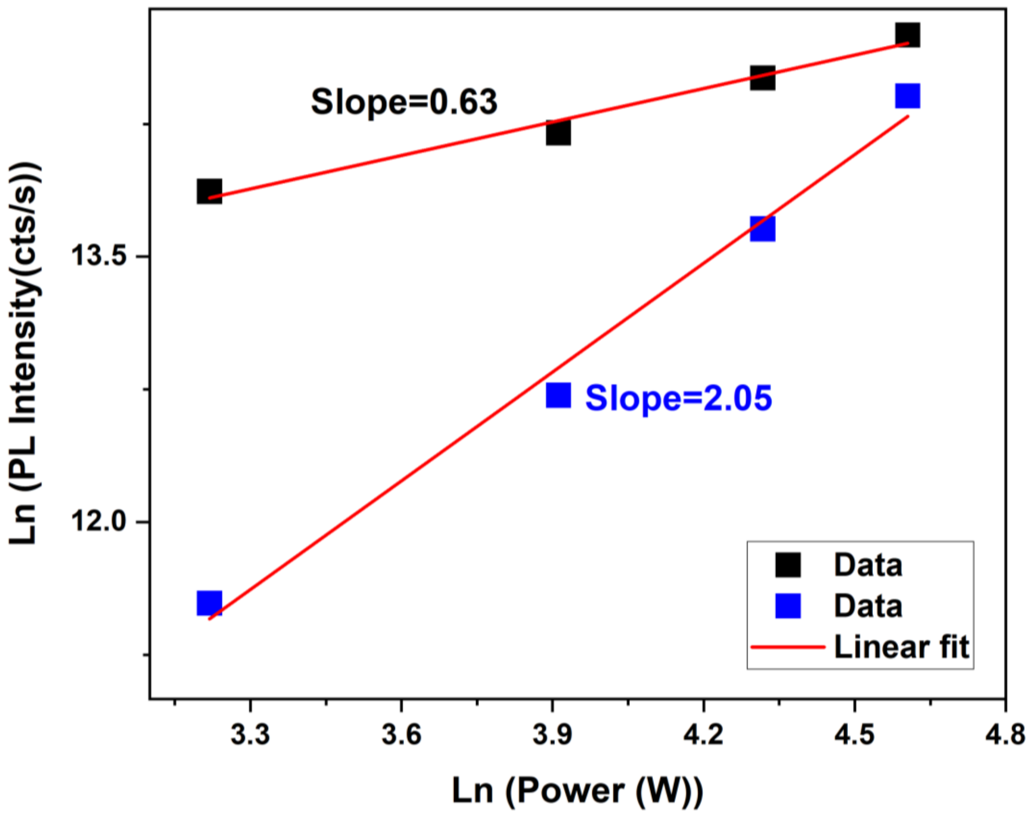

Figure S1: Power-law of the main peak (blue squares) and the low-energy peak (black squares) of κ-Ga_2O_3/GaAs heterostructure. The maximal excitation power is fixed at P_0=100 mW. Red straight lines are best fits to the data with a function of $I_{PL} = \alpha P^d$.

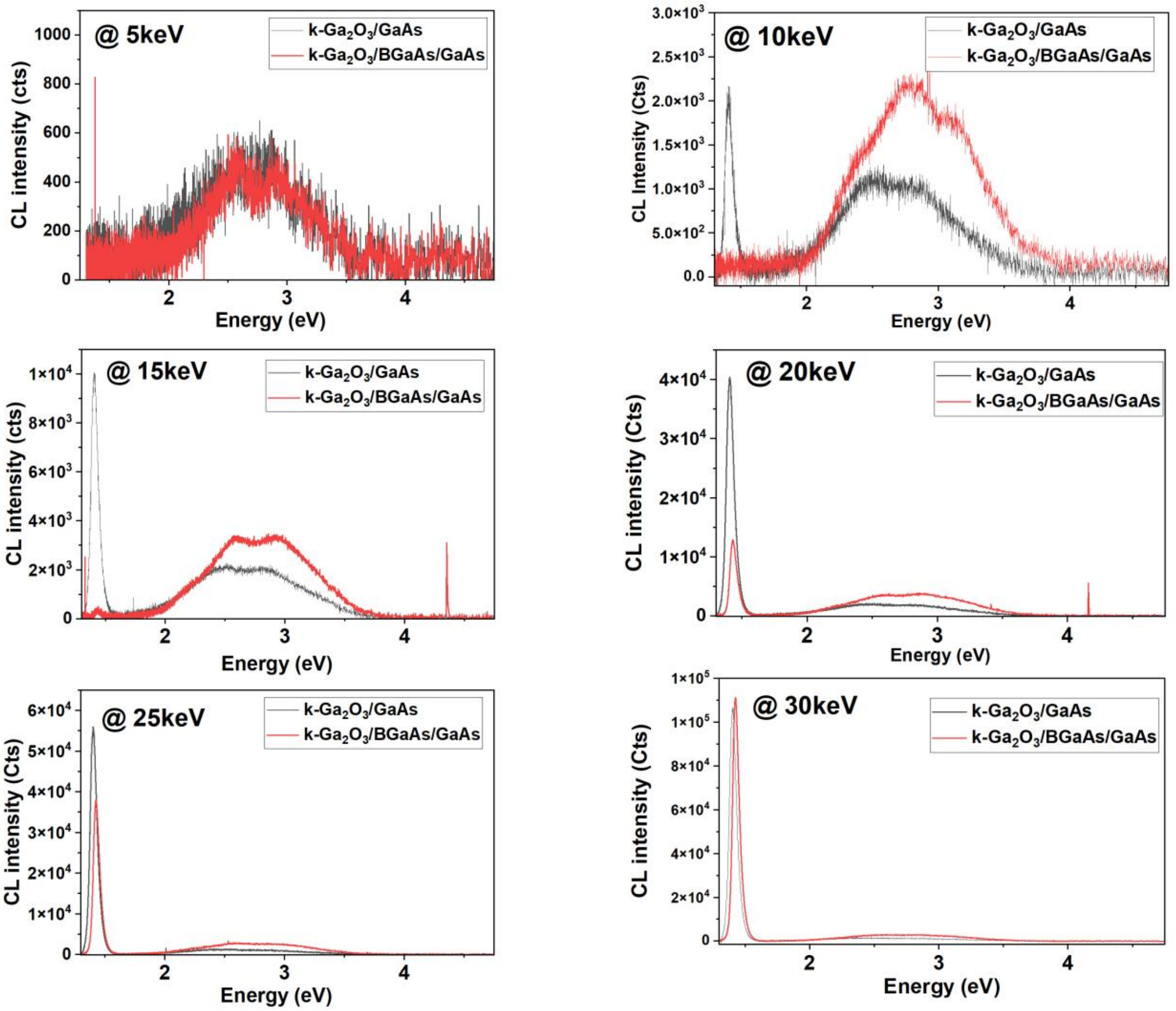

Figure S2: Comparison of the CL emission from κ-Ga_2O_3/GaAs and κ-Ga_2O_3/BGaAs/GaAs heterostructures at different e-beam energies showing the emission enhancement in the visible region.

Table S1: **Input parameters of the different PSC layers utilized for the SCAPS-1D simulation.**

Properties	Ga_2O_3 [3]	BGaAs	GaAs [4]
Thickness (µm)	0. 5	0.25 (varied)	0.5
Band gap Eg (eV)	4.8	1.25 [2]	1.42
Affinity (eV)	3.5	3.95 (varied)	4.07
Dielectric Permittivity (ϵ_r)	9.93	12.9	12.9
DOS N_C (cm^{-3})	2.2×10^{18}	4.7×10^{17}	4.7×10^{17}
DOS N_V (cm^{-3})	1.8×10^{19} or higher	7×10^{18}	7×10^{18}

Mobility μ_n ($cm^2(Vs)^{-1}$)	10 or 5	8500	8500
Mobility μ_p ($cm^2(Vs)^{-1}$)	10 or 10^{-6}	400	400
Donor density N_D (cm^{-3})	2×10^{10} - 2×10^{14}	0	0
Deep acceptor Density N_A (cm^{-3})	1×10^{10} - 1×10^{14} $N_A \approx N_D-10^{10}$	1×10^{16}	01×10^{18}
Effective defect density N_t (cm^{-3})	1×10^{14} or higher	10^{14}	1×10^{14}
Reference for the defect energy level E_t	Above Ev	Above Ev	Above Ev
Energy reference (eV)	2.23	0.6	0.6
Radiative recombination coefficient (cm^3/s)	5×10^{-11}	1.5×10^{-10}	1.5×10^{-10}
Auger electron/hole capture coefficient (cm^6/s)	1×10^{-30}	3×10^{-30}	3×10^{-30}

NOTE: The simulation is carried out with the intent of maximizing the solar cell performance, therefore it is necessary to identify a set of optimized input parameters. This is done using the results of the present investigation as well as the reliable data from existing literature reports.

Simulated data generated by varying some properties of the κ-Ga_2O_3 layer

Table S2: Variation of donor density in Ga_2O_3 and its effect on the cell performance parameters.

N_D (cm^{-3})	PCE (%)	Voc (V)	Jsc (mA/cm^2)	FF (%)
2.00000000e+10	7.95738007e+00	9.10352897e-01	3.69981979e+01	2.36254309e+01
2.00000000e+11	7.95738959e+00	9.10352829e-01	3.69981980e+01	2.36254609e+01
2.00000000e+13	7.95845575e+00	9.10354156e-01	3.69982383e+01	2.36285662e+01
2.00000000e+14	7.96816280e+00	9.10365559e-01	3.69986019e+01	2.36568575e+01
1.00000000e+15	8.01023835e+00	9.10271958e-01	3.70001733e+01	2.37832118e+01
1.00000000e+16	8.50391614e+00	9.10761138e-01	3.70129628e+01	2.52267100e+01
1.00000000e+17	1.41693897e+01	9.12810901e-01	3.70343911e+01	4.19145786e+01
1.00000000e+18	2.84065512e+01	9.13848080e-01	3.70344084e+01	8.39342243e+01
1.00000000e+19	2.85254355e+01	9.13849652e-01	3.70344094e+01	8.42853505e+01

(Other parameters were kept fixed: Thickness = 0.5μm, bandgap =4.8eV, Affinity (eV)= 3.5, Dielectric Permittivity (ϵ_r) = 9.93, DOS N_C (cm^{-3}) = 2.2 $\times10^{18}$, DOS N_V (cm^{-3}) = 1.8 $\times10^{19}$, hole mobility μ_n ($cm^2(Vs)^{-1}$) = 10, electron Mobility μ_p ($cm^2(Vs)^{-1}$) = 10, acceptor density N_A (cm^{-3}) = 0, Effective defect density N_t (cm^{-3}) = 1×10^{14})

Table S3: Variation of acceptor density of Ga_2O_3 and its effect on the cell performance parameter.

N_A (cm^{-3})	PCE (%)	Voc (V)	Jsc (mA/cm^2)	FF (%)
0.00000000e+00	2.85254355e+01	9.13849647e-01	3.70344094e+01	8.42853509e+01
1.00000000e+10	2.85254355e+01	9.13849645e-01	3.70344094e+01	8.42853512e+01
1.00000000e+11	2.85254355e+01	9.13849649e-01	3.70344094e+01	8.42853508e+01

**1.00000000e+12**	**2.85254355e+01**	**9.13849667e-01**	**3.70344094e+01**	**8.42853491e+01**
**1.00000000e+13**	**2.85254355e+01**	**9.13849650e-01**	**3.70344094e+01**	**8.42853507e+01**
**1.00000000e+14**	**2.85384500e+01**	**9.13909598e-01**	**3.70344094e+01**	**8.43182738e+01**
**1.00000000e+15**	**2.85384490e+01**	**9.13909605e-01**	**3.70344094e+01**	**8.43182703e+01**
**1.00000000e+16**	**2.85254264e+01**	**9.13849633e-01**	**3.70344094e+01**	**8.42853251e+01**
**1.00000000e+17**	**2.85253428e+01**	**9.13849571e-01**	**3.70344094e+01**	**8.42850840e+01**
**1.00000000e+18**	**2.85244104e+01**	**9.13848587e-01**	**3.70344094e+01**	**8.42824196e+01**

Because Ga_2O_3 is an n-type layer, the acceptor density value is set to 0. (Other parameters were fixed: Thickness = 0.5µm, bandgap =4.8 eV, Affinity (eV)= 3.5, Dielectric permittivity (ϵ_r) = 9.93, DOS N_C (cm^{-3}) = 2.2 ×10^{18}, DOS N_V (cm^{-3}) = 1.8 ×10^{19}, hole mobility µn ($cm^2(Vs)^{-1}$) = 10, electron mobility μ_p ($cm^2(Vs)^{-1}$) = 10, donor density N_D (cm^{-3}) = 1×10^{19}, Effective defect density N_t (cm^{-3}) = 1×10^{14}).

Table S4: Defect (deep trap centers) density variation of Ga_2O_3 layer and its effect on the cell performance parameters.

**N_t (cm^{-3})**	**PCE (%)**	**Voc (V)**	**Jsc (mA/cm^2)**	**FF (%)**
**1.00000000e+10**	**2.85254355e+01**	**9.13849659e-01**	**3.70344094e+01**	**8.42853498e+01**
**1.00000000e+11**	**2.85254355e+01**	**9.13849643e-01**	**3.70344094e+01**	**8.42853514e+01**
**1.00000000e+12**	**2.85254355e+01**	**9.13849658e-01**	**3.70344094e+01**	**8.42853500e+01**
**1.00000000e+13**	**2.85254355e+01**	**9.13849637e-01**	**3.70344094e+01**	**8.42853518e+01**
**1.00000000e+14**	**2.85254355e+01**	**9.13849652e-01**	**3.70344094e+01**	**8.42853505e+01**
**1.00000000e+16**	**2.85254355e+01**	**9.13849659e-01**	**3.70344094e+01**	**8.42853499e+01**
**1.00000000e+18**	**2.85254355e+01**	**9.13849648e-01**	**3.70344094e+01**	**8.42853509e+01**
**1.00000000e+19**	**2.85254355e+01**	**9.13849656e-01**	**3.70344094e+01**	**8.42853502e+01**

(Other parameters were fixed: Thickness = 0.5µm, band gap =4.8eV, Affinity (eV)= 3.5, Dielectric Permittivity (ϵ_r) = 9.93, DOS NC (cm^{-3}) = 2.2 ×10^{18}, DOS N_V (cm^{-3}) = 1.8 ×10^{19}, hole Mobility μ_n ($cm^2(Vs)^{-1}$) = 10 , electron Mobility μ_p ($cm^2(Vs)^{-1}$) = 10, donor density N_D (cm^{-3}) = 1×10^{19}, acceptor density N_A (cm^{-3}) = 0, Effective defect density N_t (cm^{-3}) = 1×10^{14})

Table S5: Effect of varying the electron mobility of Ga_2O_3 on the SC performances. (All other above-mentioned parameters are fixed).

**Electron mobility**	**PCE (%)**	**Voc (V)**	**Jsc (mA/cm^2)**	**FF (%)**
**1.00000000e-04**	**3.36773367e-01**	**9.13863742e-01**	**1.47703510e+00**	**2.49497078e+01**
**1.00000000e-06**	**3.37160635e-03**	**9.13864104e-01**	**1.48049317e-02**	**2.49200450e+01**
**2.00000000e+01**	**3.37160635e-03**	**9.13864104e-01**	**1.48049317e-02**	**2.49200450e+01**

Table S6: Input parameters of interface layer for SCAPS-1D simulation (default parameters for high performance).

Interface layer	Ga_2O_3/BGaAs	BGaAs /GaAs
Defect type	Neutral	Neutral
Capture cross section of electrons (cm^2)	1×10^{-19}	1×10^{-19}
Capture cross section of holes (cm^2)	1×10^{-19}	1×10^{-19}
Reference for the defect energy level E_t	Above Ev	Above Ev
Energy reference (eV)	0.6	0.6
Total density (cm^{-2})	10^{14}	10^{14}

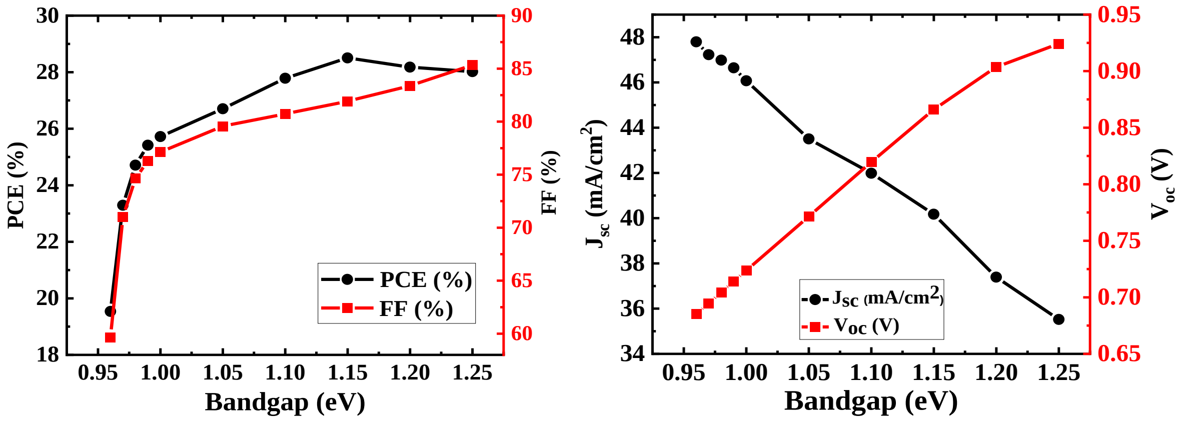

Figure S3. Effect of the BGaAs bandgap variation on the PCE, FF, J_{sc} and V_{oc} of the SC.

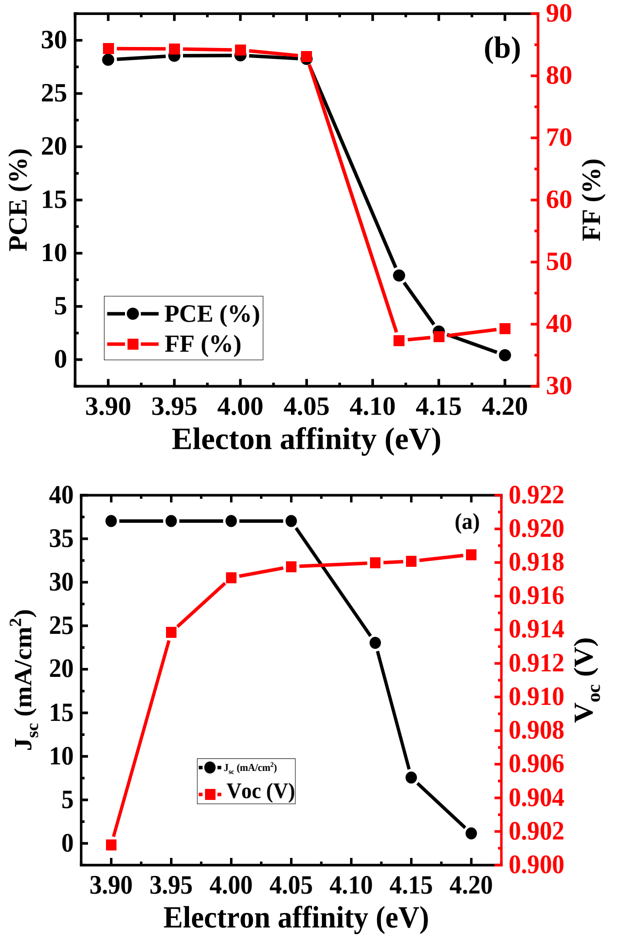

Figure S4: Analysis of the electron affinity effect in the BGaAs layer on the PCE, FF, J_{sc} and V_{oc} of the SC.

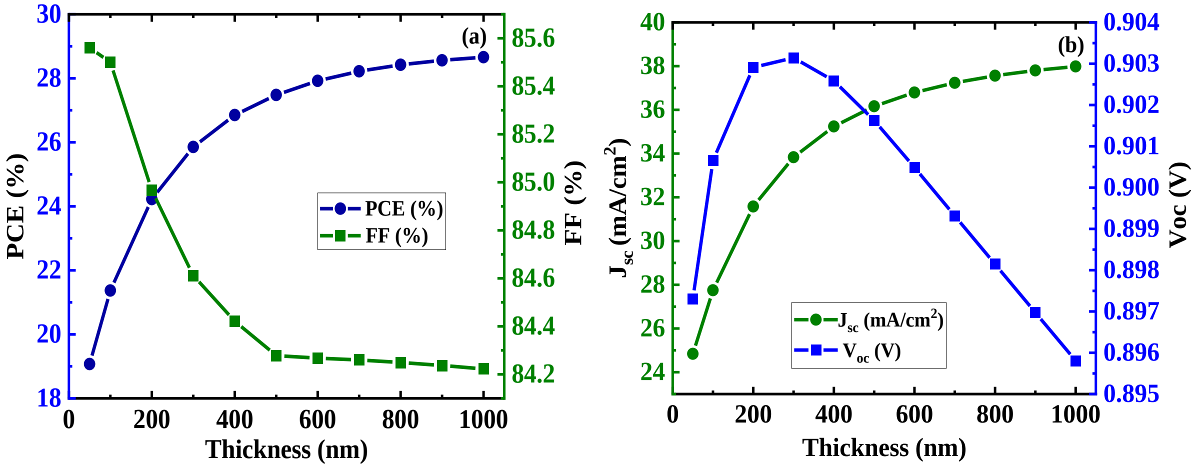

Figure S5: Effect of the BGaAs thickness on the PCE, FF, J_{sc} and V_{oc} of the SC.

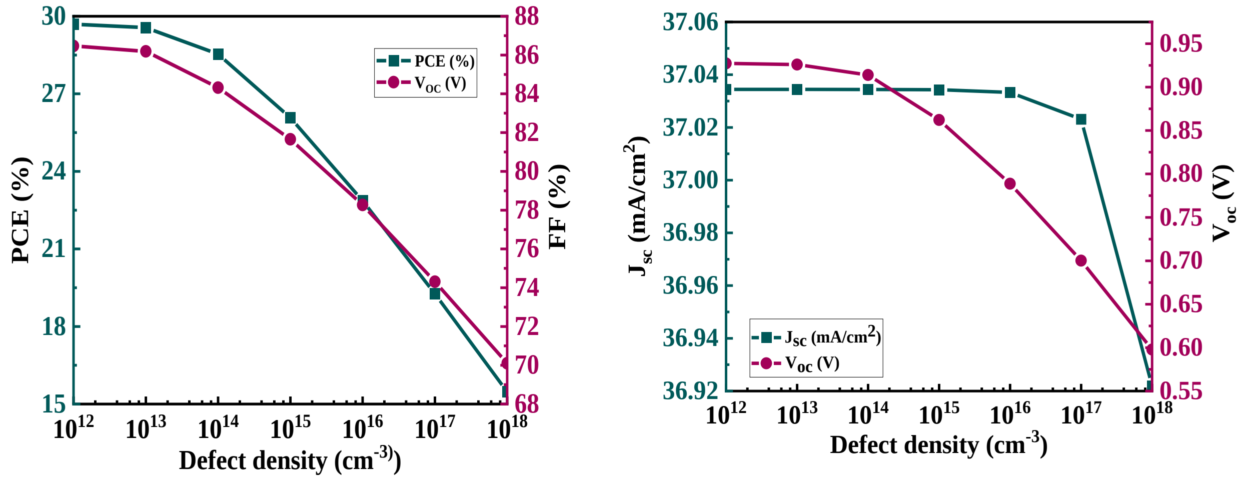

Figure S6: Effect of the bulk defect density in the BGaAs layer on the PCE, FF, J_{sc} and V_{oc} of the SC.

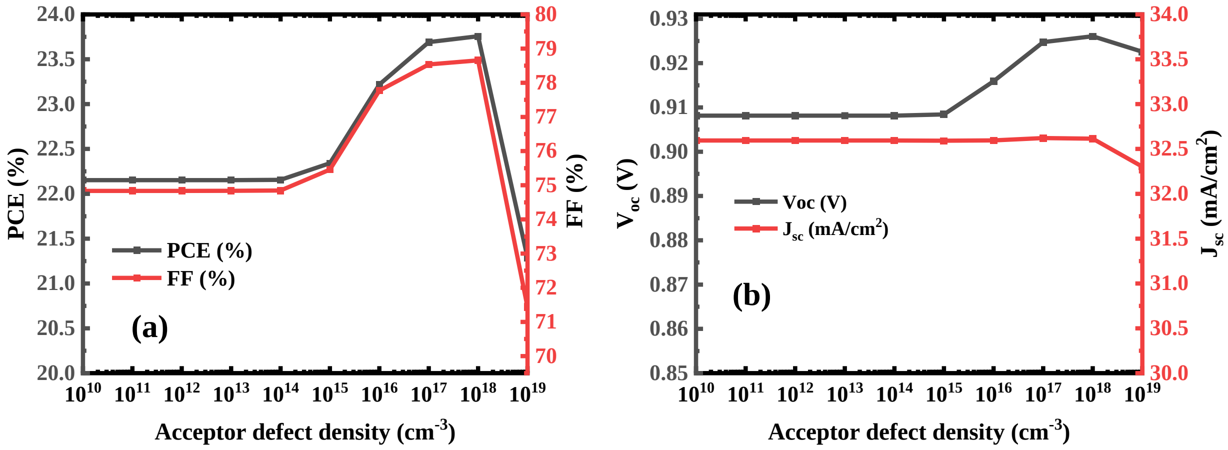

Figure S7. Effect of the acceptor defect density in the BGaAs layer on the (a) PCE and FF, (b) J_{sc} and V_{oc} of the SC.

References

[1] Adnan Alwan Mouhammed, Ayed N. Saleh, Simulation Effect of Ga_2O_3 layer thickness on CdTe solar cell by SCAPS-1D, Tikrit Journal of Pure Science, Tikrit Journal of Pure Science, (2019) 24, 110/116.

[2] Hidouri Tarek, Mahitosh Biswas, Indranil Mal, Samia Nasr, Subhananda Chakrabarti, Dip Prakash Samajdar, and Faouzi Saidi. "Engineering of carrier localization in BGaAs SQW for novel intermediate band solar cells: Thermal annealing effect." Solar Energy, (2020) 199, 183-191.

[3] Mohammed Azza, El Hadi Chahid, Abdellatif Hmairrou, Rachid Abdia, Malika Tridane, Abdessamad Malaoui, Said Belaaouad, Numerical Simulation of p-i-n GaAs Photovoltaic Cell Using SCAPS-1D, J. Biointerface research in applied chemistry, (2023) 13, 253.
